# Oral mucosal melanoma: a malignant trap

**DOI:** 10.1186/1746-160X-2-7

**Published:** 2006-03-28

**Authors:** Emmanouil K Symvoulakis, Dionysios E Kyrmizakis, Emmanouil I Drivas, Anastassios V Koutsopoulos, Stylianos G Malandrakis, Charalambos E Skoulakis, John G Bizakis

**Affiliations:** 1 Department of Otorhinolaryngology, University of Crete School of Medicine, Heraklion, Crete, Greece; 2 Pathology Department, University of Crete School of Medicine, Heraklion, Crete, Greece

## Abstract

Oral mucosal melanomas are highly malignant tumors. The 'chameleonic' presentation of a mainly asymptomatic condition, the rarity of these lesions, the poor prognosis and the necessity of a highly specialized treatment are factors that should be seriously considered by the involved health provider. We present the case of a 75-year-old man who was referred to the Ear, Nose and Throat department. His symptoms were voice alteration and saliva drooling, progressively worsening during the last few weeks. The absence of pain was the reason for the delay of seeking medical care. The diagnosis was an oversized oral melanoma. This is an example of how the time of diagnosis and the evolution of a disease could be seriously influenced by patient's behavior. Melanomas arising from oral mucosa have poor prognosis unless they are discovered and treated early. The vigilance of the physicians is necessary to have success in this difficult task.

## Background

Primary mucosal melanomas of the head and neck are a rare entity, occurring much less frequently than their cutaneous counterparts. Among those of the head and neck region, oral mucosal melanoma is extremely infrequent. It accounts for only 0.5% of oral neoplasms [1]. Oral mucosal melanomas are highly malignant tumors with the tendency to metastasize or locally invade tissues more readily than other malignant tumors of the oral cavity [1].

One third of the patients are asymptomatic at the time of diagnosis and episodes of hemorrhage seem to be the leading symptom [2].

We present the case of a 75-year-old man who was referred to the Ear, Nose and Throat department having symptoms as voice alteration and saliva drooling, progressively worsening. The diagnosis was an oversized palate melanoma blocking the oral cavity. In this case, the delay in seeking medical care was probably due to the 'silent' course of the disease in relation to the patient's tendency to underestimate his symptoms.

## Case presentation

A 75-years-old male during an ordinary visit for drug prescription due to his chronic health problems, described his complaints (voice alteration, dysphagia and saliva drooling progressively worsening during the last few weeks) and his general physician referred him for an E.N.T. evaluation. The lack of any pain sensation was the reason for the delay of seeing a physician. His medical history was significant for severe benign prostatic hyperplasia, hypertension and chronic obstructive pulmonary disease. He was heavy tobacco smoker until recently. No history of alcohol intake was referred.

**Figure 1:**
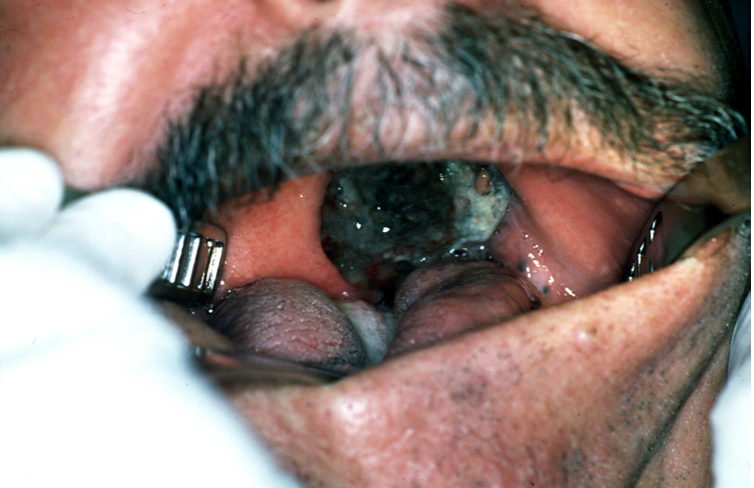
Photograph at initial examination showing an oversized pigmented soft mass (melanoma) arising from the palate.

Clinical examination of the oral cavity revealed an oversized pigmented soft mass arising from the left half of his hard palate involving the soft palate and the ipsilateral palatine tonsil causing partial obstruction of the oral cavity and the oropharynx (Figure 1). Some pigmented macules of various sizes were also noted growing at the periphery of the tumor. During neck palpation a 2 × 3 cm firm mobile non-tender mass was palpated at the left upper jugular region. A punch biopsy of the oral mass was performed.

A complete blood cell count, biochemical profile, and urinalysis were ordered without significant findings. A chest x ray was normal.

**Figure 2:**
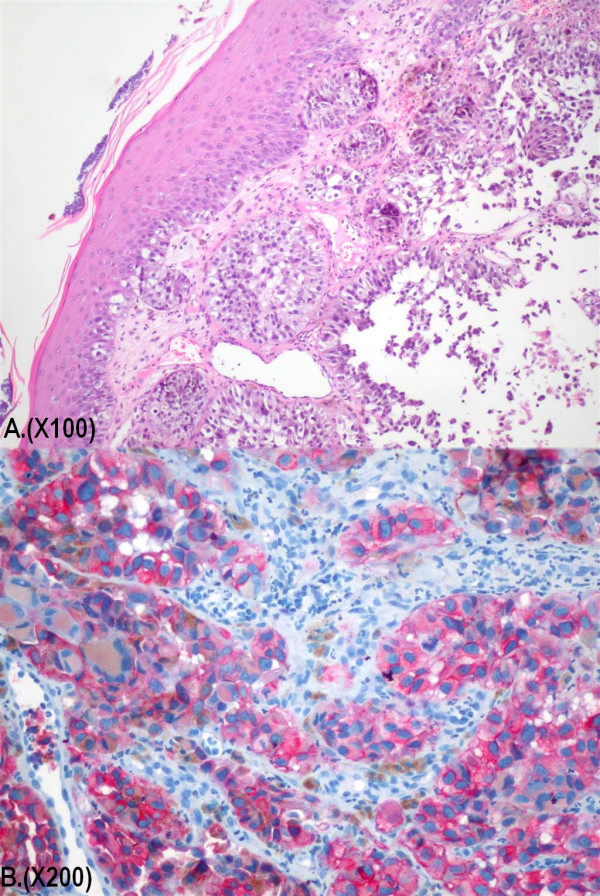
Tissue sections revealed extensive infiltration of the mucosa by neoplastic predominantly epithelioid cells, in a solid, nested, trabecular or alveolar pattern. Continuity of the tumor with the surface epithelium was identified (Haematoxylin and Eosin stain, magnification ×100) (A). The neoplastic cells demonstrate strong immunoreactivity for Melan A monoclonal antibody (magnification ×200) (B).

Histological examination of the specimen demonstrated extensive infiltration of the ulcerated mucosa by neoplastic predominantly epithelioid cells, in a solid, nested, trabecular or alveolar pattern. The cells were round to oval, having prominent eosinophilic nucleoli and atypical mitotic figures. In areas with intact surface epithelium was identified continuity of the tumor with the epithelium. There were also brown pigment deposition and neoplastic giant cells (figure 2).

**Figure 3:**
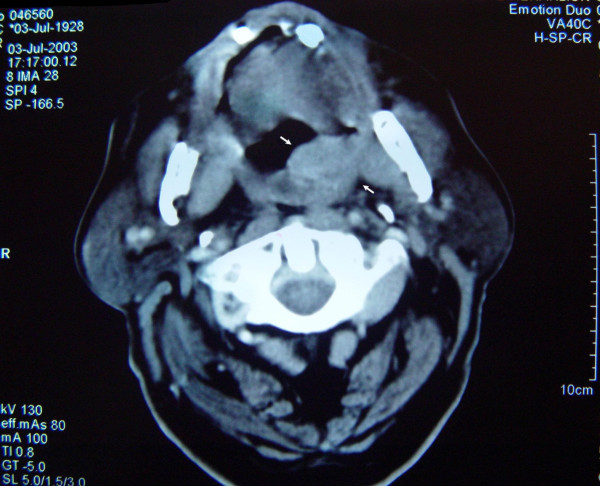
A computer tomography with contrast revealed a large palate's mass, which is seemed to infiltrate the left medial pterygoid muscle.

A computer tomography with contrast revealed pathologically enlarged cervical lymph nodes of the left neck. Signs of infiltration of the ipsilateral medial pterygoid muscle were also observed (figure 3).

Work up for distant metastases (CT scan of chest, brain and abdomen plus bone scintigraphy) was negative.

Medical information was provided to the patient and his family regarding the diagnosis, staging, therapeutic options and prognosis. Our patient excluded any option of invasive treatment. He treated for palliation by hypofractionated radiotherapy. The patient died 7 months later from cancer related cachexia.

## Discussion

Oral melanomas may be presented with a variety of morphologic and macroscopic characteristics [2,3] that could render the clinical diagnosis extremely difficult. The differential diagnosis includes melanotic macule, smoking-associated melanosis, post-inflammatory pigmentation, medication induced melanosis, melanoplakia, melanoacanthoma, nevi, Addison's disease, Peutz-Jeghers syndrome, amalgam tattoo, Kaposi's sarcoma and many other conditions sharing some macroscopic characteristics [4]. Oral amelanotic melanomas are rare and the prognosis is poorer than that of pigmented melanomas because of delays in establishing the correct diagnosis and in the initiation of treatment. The differential diagnosis of amelanotic melanoma includes poorly differentiated carcinoma and lymphoma [5]. In general oral mucosal melanomas have a poor prognosis. Common sites of metastasis are lymph nodes, liver and lung with widespread involvement occurring in advanced disease [6]. While the recommended treatment is the ablative surgery with tumor-free margins in combination with chemotherapy and, to a lesser extent, immunotherapy or irradiation, there is a recognized need for an evidence-based treatment protocol choice but probably, multimodal therapy may be proven more effective in the treatment of oral mucosal melanoma [1,7].

The morphologic 'chameleonic' presentation of a mainly asymptomatic condition, the rarity of these lesions, the poor prognosis and the necessity of a highly specialized treatment are factors that could influence the diagnostic and management process of these malignancies.

In the case reported, the patient's underestimation of his symptoms, probably due to their unpainful character, was the main reason of not seeking medical care in time. The patient described his complaints during an ordinary drug prescription visit and only then he was referred for further evaluation.

The main contribution of this case report is the reminder that because many different disciplines are examining and dealing with oral cavity lesions (which can be of great significance but less or more without symptoms) and this part of body is well accessible to the physician, the proper in details clinical examination of the oral and oropharyngeal mucosa must be performed in all patients.

In conclusion a high level of suspicion, careful history and a thorough physical examination including the oral cavity and the neck, from the part of the involved health providers being otorhinolaryngologists, general practitioners, internists or dentists regarding these malignancies are essential. Minimal or atypical signs and symptoms must be seriously considered.

Sometimes the patients have the tendency to minimize or ignore their complaints for a variety of reasons. This fact could influence the evolution of a disease. This case is an example of how the time of diagnosis and the evolution of a disease could be seriously influenced by patient's behavior. The frequency and outcomes of this phenomenon may reflect, indirectly, the quality of health care provision.

## Competing interests

The author(s) declare that they have no competing interests.

## Authors' contributions

E.K.S, D.E.K and E.I.D have drafted and prepared the manuscript. A.V.K carried out the histological evaluation. S.G.M carried out the review of the patient's medical record in order to collect all the available information. C.E.S and J.G.B were involved in revising the article for intellectual content details. All authors read and approved the final manuscript.

## References

[B1] Garzino-Demo P, Fasolis M, Maggiore GM, Pagano M, Berrone S (2004). Oral mucosal melanoma: a series of case reports. J Craniomaxillofac Surg.

[B2] Lopez-Graniel CM, Ochoa-Carrillo FJ, Meneses-Garcia A (1999). Malignant melanoma of the oral cavity: diagnosis and treatment experience in a Mexican population. Oral Oncol.

[B3] Tanaka N, Mimura M, Ichinose S, Odajima T (2001). Malignant melanoma in the oral region: ultrastructural and immunohistochemical studies. Med Electron Microsc.

[B4] Hicks MJ, Flaitz CM (2000). Oral mucosal melanoma: Epidemiology and pathobiology. Oral Oncol.

[B5] Notani K, Shindoh M, Yamazaki Y, Nakamura H, Watanabe M, Kogoh T, Ferguson M, Fukuda H (2002). Amelanotic malignant melanomas of the oral mucosa. British Journal of Oral and Maxillofacial Surgery.

[B6] Barker BF, Carpenter WM, Daniels TE, Kahn MA, Leider AS, Lozada-Nur F, Lynch DP, Melrose R, Merrell P, Morton T, Peters E, Regezi JA, Richards SD, Rick GM, Rohrer MD, Slater L, Stewart JC, Tomich CE, Vickers RA, Wood NK, Young SK (1997). Oral mucosal melanomas: the WESTOP Banff workshop proceedings. Western Society of Teachers of Oral Pathology. Oral Surg Oral Med Oral Pathol Oral Radiol Endod.

[B7] Manolidis S, Donald PJ (1997). Malignant mucosal melanoma of the head and neck: Review of the literature and report of 14 patients. Cancer.

